# Willing or Hesitant? A Socioeconomic Study on the Potential Acceptance of COVID-19 Vaccine in Japan

**DOI:** 10.3390/ijerph18094864

**Published:** 2021-05-02

**Authors:** Yoshihiko Kadoya, Somtip Watanapongvanich, Pattaphol Yuktadatta, Pongpat Putthinun, Stella T. Lartey, Mostafa Saidur Rahim Khan

**Affiliations:** 1 School of Economics, Hiroshima University, Higashi-Hiroshima 739-8525, Japan; ykadoya@hiroshima-u.ac.jp (Y.K.); somtip.w@gmail.com (S.W.); d206295@hiroshima-u.ac.jp (P.Y.); putthinun@gmail.com (P.P.); 2 Department of Epidemiology and Biostatistics, Indiana University School of Public Health, Bloomington, IN 47405, USA; stlartey@iu.edu

**Keywords:** vaccine, COVID-19 pandemic, socioeconomic factors, Japan

## Abstract

The worldwide COVID-19 vaccination program is already underway, raising hopes and aspirations to contain the spread of the COVID-19 pandemic that halted economic and social activities. However, the issue of vaccine effectiveness and its side-effects is influencing the potential acceptance of vaccines. In this uncertain situation, we used data from a nationwide survey in Japan during February 2021, following the Japanese government’s initial phase of COVID-19 vaccination. Our results show that 47% of the respondents are willing to take a vaccine once it is available, while 22% are not willing and another 31% remain indecisive. Our ordered probit regression results show that demographic, socioeconomic, and behavioral variables such as gender, age, subjective health status, children, household income, household assets, financial literacy, future anxiety, and myopic view of the future are associated with willingness to take a COVID-19 vaccine. Our findings suggest that Japan’s government should not adopt a one-size-fits-all policy to promote the vaccination program, but rather target people with specific socioeconomic backgrounds who are less willing and more hesitant to take a vaccine.

## 1. Introduction

The current novel coronavirus disease (COVID-19) is highly contagious. As of 18 March 2021—a year after the WHO categorized the COVID-19 outbreak as a pandemic, there were 120,915,219 cumulative cases and 2,674,078 total deaths [1]. The number of infected cases is believed to be underreported because of asymptomatic infection, hesitancy to get tested, and lack of alternative testing procedures [2,3,4,5]. The pandemic disrupted many aspects of human activity, leading to worldwide economic challenges including increased unemployment, income, food, and housing insecurity, and intimate partner violence [6,7,8]. In response to the crisis and to bring human activity closer to normal, institutions implemented several preventive measures, in addition to the swift development of several vaccines—with this being the fastest vaccine development in history [9,10]. Although achieving herd immunity through vaccination is considered vital in reducing the spread of COVID-19 and restoring normalcy in human activities, public perceptions of COVID-19 vaccines are still mixed [11,12,13,14,15]. These perceptions will likely affect the willingness to receive the vaccines, lead to lower vaccination rates, and put the unvaccinatable vulnerable groups at greater risk.

Previous studies show that public willingness to receive a COVID-19 vaccine varies worldwide and over time. Lazarus et al. conducted a survey in June 2020 and found that, if the vaccines are proven to be safe and effective, 75.4% of respondents in the US, and 54.8% to 88.6% of respondents worldwide are willing to be vaccinated [12]. In contrast, Guidry et al.’s July 2020 survey [11] revealed that only around 60% of their sample were probably or certainly planning to get a vaccination. Sallam [16] and Al-Qerem and Jarab [17] reported even lower vaccination acceptance rates in the Middle East, US, Russia, Africa, and several European countries. The gap in willingness to vaccinate between these studies emphasizes the nature of public perceptions of vaccination. Some studies found correlations and/or causation between vaccine hesitancy and skepticism over vaccine safety, possible side-effects, and effectiveness [11,17,18,19,20,21,22,23,24]. Moreover, Sallam et al. [25] attributed vaccine hesitancy to misinformation and conspiracy beliefs such as injecting microchips and infertility. Hence, this willingness may have changed over time, especially during the vaccination’s actual rollout as people see reports of the vaccine efficacy rate, mild side-effects such as allergic reactions, and further vaccine safety tests [26,27,28,29,30,31]. In Japan, although an earlier survey in September 2020 found that approximately 66% of Japanese were willing to vaccinate [23], public willingness has changed since the receipt of the first vaccine in December 2020 [32,33]. Machida et al. [33], who conducted a survey from 14 to 18 January 2021, found that approximately 62% of Japanese are more willing to receive COVID-19 vaccination, as reported vaccine allergy cases started to appear in the Japanese media when vaccination in the US and UK started [34,35]. Since then, more cases continued to emerge after vaccination for Japanese frontline medical staff began in February 2021 [36,37,38]. Japan has one of the lowest levels of trust and confidence in vaccinations in general [39]; hence, the people are less likely to be willing to receive COVID-19 vaccination. This skepticism may, thus, jeopardize the prospects of achieving the needed herd immunity in Japan.

As Japan will soon commence its first mass public vaccination, a deep understanding of public willingness to receive a COVID-19 vaccination and public vaccine hesitancy is vital. This is necessary for several reasons, including public policy development, education targeting, advocacy, and expectation and outcome management. Thus, this study examines the association between socioeconomic factors and people’s willingness and/or hesitancy to receive a COVID-19 vaccination. Although previous studies investigated vaccination willingness [23,40] or the relationships between perspectives of vaccination and socioeconomic factors [33,41,42,43,44], these studies were conducted before clinical trials were completed in November 2020 [45] or before the initial distribution of the COVID-19 vaccine in Japan [46]. We conducted our survey in February 2021, after the initial distribution of COVID-19 vaccines in several countries, including the US and UK, and during the initial vaccine distribution among essential medical workers in Japan. Hence, our survey is timelier and reflects attitudes when the population is actually facing the vaccine rollout and challenges regarding reactions, distribution, and development. Moreover, we can observe people’s willingness to be vaccinated after seeing the vaccination’s side-effects in Japan and other countries. Thus, our study is more likely to provide a timely prediction of the willingness to be vaccinated and vaccine hesitancy.

To the best of our knowledge, this study is the first to report empirical evidence on the level of willingness to receive a COVID-19 vaccine after its initial distribution and to explore its association with the different socioeconomic factors in the Japanese population. Thus, this study holds vital lessons for policymakers in Japan and other countries to identify the characteristics of those who are unwilling to receive COVID-19 vaccination and implement effective policy interventions, which will ultimately help them achieve herd immunity, protect the vulnerable, and subsequently restore normalcy in human and economic activities.

The rest of the paper is organized as follows: Section 2 presents the data and methodology. We report the empirical results in Section 3 and the discussion in Section 4. Section 5 concludes the manuscript.

## 2. Materials and Methods

### 2.1. Data

Our study uses panel data from the Household Behavioral and Financial Survey funded by Hiroshima University’s Hiroshima Institute of Health Economics Research (HiHER). The panel survey was conducted by Nikkei Research, a leading research company in Japan. Nikkei Research’s database is one of the largest in the country and representative of Japanese population from all socioeconomic backgrounds. The first online survey was conducted around the beginning of the COVID-19 pandemic from 20–25 February 2020 using a random sampling procedure. A year later, the second online survey was conducted around the beginning of the COVID-19 vaccination program in Japan from 19–26 February 2021 and targeted individuals who responded to the 2020 survey. The datasets consist of information on socioeconomic characteristics and preferences for the population in Japan aged 20 and above. It had 17,463 and 6103 total observations in 2020 and 2021, respectively.

We utilized the 2021 dataset, which contains respondents’ socioeconomic status and questions regarding willingness to receive COVID-19 vaccination. Additionally, we used demographic characteristics and preferences, including gender, age, education, place of residence, children in the household, and financial literacy from the 2020 dataset. Our final sample consisted of 4253 observations or approximately 70% of total observations in the 2021 dataset. Regrettably, we had to exclude 1850 observations due to missing data on important socioeconomic variables such as household income and household assets.

### 2.2. Variable Definitions

Our dependent variables “willingness to take vaccine” and “vaccine hesitancy” were based on the 2021 survey’s item “Once the COVID-19 vaccine becomes available free of charge, I will take it soon” rated on the five-point scale described in detail in Table 1. We treated “willingness to take vaccine” as an ordinal variable on the basis of the above item and “vaccine hesitancy” as a binary variable following Fisher et al. [20].

For the explanatory variables, we included gender, age, education, place of residence, marital status, children in the household, living situation, employment status, household income, and household assets as demographic variables. We also included financial literacy as a proxy for rational decision-making ability in health-related behaviors, as suggested by [47,48,49,50,51]. Furthermore, we included subjective health status, future anxiety, risk preference, and myopic view of the future. Table 1 provides the definitions of all the variables.

### 2.3. Descriptive Statistics

Table 2 presents the descriptive statistics of the sample, which contained 4253 observations. The average score of willingness to take vaccine was 3.41 out of 5. Additionally, 53% of the sample exhibited some vaccine hesitancy. To provide further detail, Table 3 and Table 4 present the distribution of the willingness to take vaccine and vaccine hesitancy variables classified by age group. The results in Table 3 show that, overall, most of the respondents (1333 observations or 31.34% of the total sample) answered 3 for the statement “Once the COVID-19 vaccination becomes available with free of charge, I will take it soon”, followed by 5 at 25.11%, 4 at 21.51%, 2 at 13.28%, and 1 at 8.75%. If we divide the sample into five age groups, the majority of respondents in each age group answered 3 except the respondents in oldest age group, who answered 5, implying that they are the most willing to take the vaccine (37.68% of the sample in the oldest age group). The results in Table 4 for vaccine hesitancy show that the vaccine hesitancy rate was highest in respondents aged 31–40 and tended to decrease as the respondents became older—from 62.32% in the sample aged 31–40 to 35.75% in the sample aged 61 and above.

For the demographic variables, Table 2 shows that about 65% of respondents were men and the average age was 50.32 years. Approximately 62% of the sample held a university degree and 61% of the sample lived in a central area (around the Tokyo or Osaka metropolitan areas). For household status, 66% of the sample were married, 57% had children, and 20% lived alone. About 64% of the respondents were employed. Respondents had annual household incomes of approximately 6.34 million JPY on average and 19.80 million JPY in household assets in 2021. The average financial literacy score was 0.65; that is, they could answer wo out of the three financial literacy questions correctly. On average, respondents rated their subjective health status, future anxiety, and myopic view of the future at 3.24, 3.71, and 2.69, respectively, out of 5. Overall, the respondents had risk preferences of 46%; in other words, they were slightly risk-averse.

### 2.4. Methodology

We explored the association between socioeconomic factors and respondents’ willingness to take vaccine and vaccine hesitancy using Equations (1) and (2), respectively.

(1) $$Y_{i1}=f\left(X_{i},\varepsilon_{i}\right),$$

(2) $$Y_{i2}=f\left(X_{i},\varepsilon_{i}\right),$$

where $Y_{1}$ is willingness to take vaccine, $Y_{2}$ is vaccine hesitancy, X is a vector of individual characteristics, and ε is the error term. We used an ordered probit (oprobit) regression to estimate Equation (1), as the dependent variable was an ordinal variable, and a probit regression to estimate Equation (2), as the dependent variable was a binary variable. Since the probit model estimates the probability of an observation to fall into one of the specified categories, we believed that probit models were suitable to predict the probability of willingness (or hesitancy) of respondents to take COVID-19 vaccine.

As there was potential multicollinearity between the explanatory variables in the models (i.e., individuals with a high level of education could have high financial literacy), we conducted correlation and multicollinearity tests in all models (provided as Supplementary Materials). The correlation matrix showed a weak relationship between the explanatory variables (lower than 0.70). In addition, the variance inflation factor tests of the explanatory variables were below 10, indicating that multicollinearity was not significant in all models.

Equations (3) and (4) provide the full model specifications of Equations (1) and (2), respectively.

(3) $$\begin{array}{l} Willingness\;to\;take\;vaccine_{i}=\beta_{0}+\beta_{1}male_{i}+\beta_{2}age_{i}+\beta_{3}age\;squared_{i}+\\ \beta_{4}university\;degree_{i}+\beta_{5}live\;in\;central\;area_{i}+\beta_{6}marriage_{i}+\beta_{7}children_{i}+\\ \beta_{8}live\;alone_{i}+\beta_{9}employed_{i}+\beta_{10}log\;of\;household\;income_{i}+\\ \beta_{11}log\;of\;household\;assets_{i}+\beta_{12}financial\;literacy_{i}+\beta_{13}subjective\;health_{i}+\\ \beta_{14}future\;anxiety_{i}+\beta_{15}level\;of\;risk\;preference_{i}+\\ \beta_{16}myopic\;view\;of\;the\;future_{i}+\varepsilon_{i}. \end{array}$$

(4) $$\begin{array}{l} Vaccine\;hesitancy_{i}\;=\beta_{0}\;+\beta_{1}male_{i}+\beta_{2}age_{i}+\beta_{3}age\;squared_{i}+\\ \beta_{4}university\;degree_{i}+\beta_{5}live\;in\;central\;area_{i}+\beta_{6}marriage_{i}+\beta_{7}children_{i}+\\ \beta_{8}live\;alone_{i}+\beta_{9}employed_{i}+\beta_{10}log\;of\;household\;income_{i}+\\ \beta_{11}log\;of\;household\;assets_{i}+\beta_{12}financial\;literacy_{i}+\beta_{13}subjective\;health_{i}+\\ \beta_{14}future\;anxiety_{i}+\beta_{15}level\;of\;risk\;preference_{i}+\\ \beta_{16}myopic\;view\;of\;the\;future_{i}+\varepsilon_{i}. \end{array}$$

## 3. Empirical Results

We first report the results of the full sample analysis in Section 3.1. In Table 5 and Table 6, we report the results when using the willingness to take vaccine and vaccine hesitancy as the dependent variables, respectively. We then performed the same analysis after dividing the sample by age and gender in Section 3.2 and Section 3.3, respectively.

Each table below presents the results of four specifications of the explanatory variables. The first specification includes controls for only the demographic variables. In the second specification, we added financial variables including household income, household assets, and financial literary. The third specification includes respondents’ subjective health status and future anxiety ratings. Lastly, the fourth specification includes respondents’ risk preferences and myopic views of the future.

### 3.1. Full Sample Analysis

In Table 5, the ordered probit model regression results show that male, age squared, university degree, having children, employment, log of household income, log of household assets, financial literacy, subjective health status, and future anxiety had a positive and significant impact on the willingness to take vaccine across the models (except for university degree, employment, and log of household assets, which showed a significant impact in some models). In contrast, only one variable, age, showed a negative and strongly significant impact on the willingness to take vaccine across the models.

In Table 6, in which we used vaccine hesitancy as the dependent variable, the results from the probit model regression were consistent with the results in Table 5. Most of the variables that showed a significant impact in Table 5 also showed a significant impact in Table 6, albeit in opposite directions. In Table 6, male, age squared, children, log of household assets, financial literacy, subjective health status, and future anxiety had negative and strongly significant impacts, whilst age had a positive and strongly significant impact on vaccine hesitancy in the final specification (Model 2.4). In addition to the results in Table 5, level of risk preference showed a positive and weakly significant impact at the 10% level on vaccine hesitancy in Model 2.4.

### 3.2. Subsample Analysis by Age

**Figure 1:**
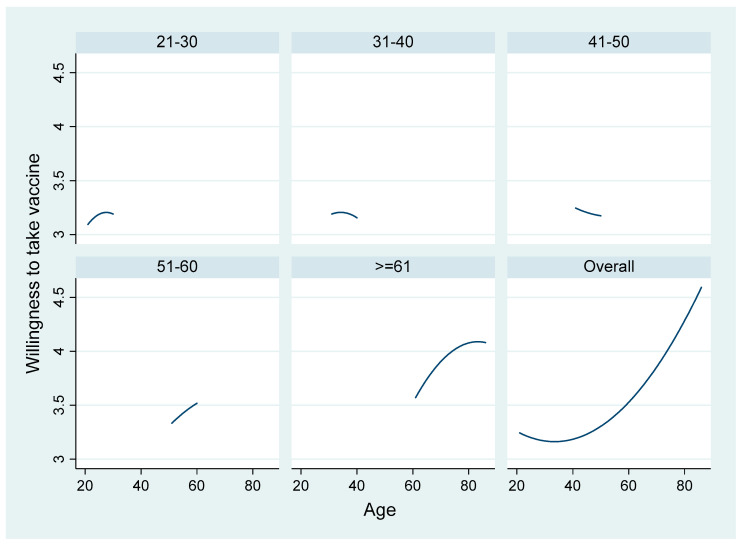
Fitted values of the willingness to take vaccine variable by age group.

**Figure 2:**
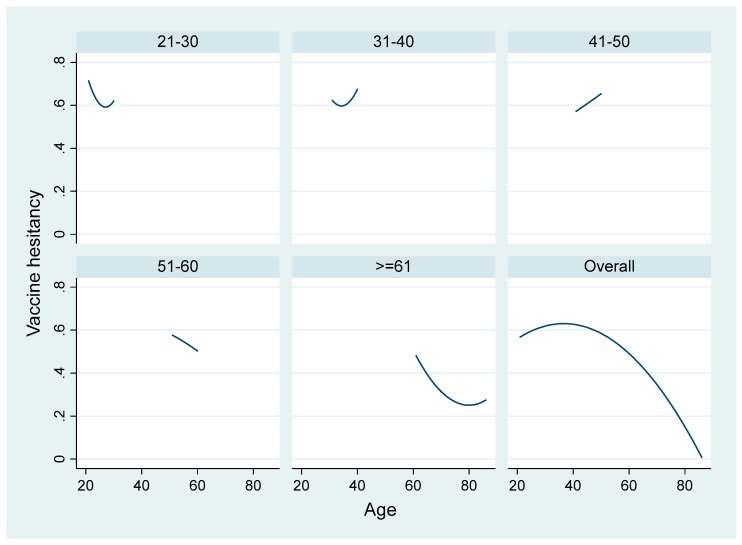
Fitted values of vaccine hesitancy variable by age group.

Figure 1 and Figure 2 show that age had a nonlinear relationship with willingness to take vaccine and vaccine hesitancy; hence, we further explored the impact of age in the subsample analysis. We divide the respondents by age into two groups, respondents aged under 65 and respondents aged 65 and above, and we applied the same estimation methods. Table 7 reports the results from the ordered probit model regression and Table 8 shows the results from the probit model regression.

The results in Table 7 and Table 8 show that, overall, there were no differences in the significance of the estimated parameters compared to the results in Table 5 and Table 6. Most of the coefficients had consistent signs and significance levels across models and specifications, except the myopic view of the future variable, which became significant in Table 7 compared to Table 5. The results for the subsample aged under 65 (Models 3.1–3.4 and 5.1–5.4) were similar to the full sample results (Models 1.1–1.4 and 2.1–2.4) since this group of respondents represented the majority of our sample (3485 observations or 82% of the full sample). However, the results for the subsample aged over 65 (Models 4.1–4.4 and 6.1–6.4) indicate that only being male, having children, and the log of household assets still had a significant impact on the willingness to take vaccine (positive coefficients) and vaccine hesitancy (negative coefficients).

### 3.3. Subsample Analysis by Gender

The results of full sample analysis in Table 5 and Table 6 indicated that male respondents were more willing to take vaccine and exhibited less vaccine hesitancy. Therefore, we further explored the impact of gender in the subsample analysis and report the results in Table 9 and Table 10.

Overall, the results in Table 9 and Table 10 were similar to the full sample analysis in Table 5 and Table 6. The age, age squared, children, and subjective health variables showed significant effects on the willingness to take vaccine and vaccine hesitancy in both the male and the female subsamples in Table 9 and Table 10. Comparing the male and female subsamples, the log of household assets, financial literacy, future anxiety, and myopic view of the future had significant impacts in the male subsample (Models 8.2–8.4 and 10.2–10.4), while the log of household income had a significant impact in the female subsample (Models 7.2–7.4 and 9.2–9.4).

## 4. Discussion

Our findings show that willingness to take vaccine and vaccine hesitancy in Japan changed over time. Yoda and Katsuyama [23] and Machida et al. [33] reported that 65.7% and 62.1% of Japanese were willing to be vaccinated and that 34.27% and 37.9% were hesitant or would refuse vaccination, respectively. After the vaccination program’s deployment in Japan from February 2021, 46.62% of Japanese were willing to be vaccinated (Table 3). Approximately 53% were hesitant or would refuse vaccination (Table 4). These changes may support our conjecture that news about the vaccines affects the Japanese people’s attitudes toward vaccination, as mentioned earlier.

We obtained several significant findings from the full model specification (Model 1.4 in Table 5 and Model 2.4 in Table 6), some of which are consistent with prior studies in Japan [23,33], while others are not. The effects of gender, for example, are similar to those reported in [23,33]. We found that men had a higher willingness and were less hesitant to receive vaccination than women. This hesitancy stems from women’s concerns over the COVID-19 vaccine’s potential side-effects, as presented in [23]. This concern among women also may be linked to earlier safety concerns in Japan over other vaccines such as the human papillomavirus (HPV) vaccine [39]. Prior studies also found similar results before the deployment of mass vaccination programs in other countries [17,52,53]. On the other hand, the effects of age, subjective health status, and having/not having children are inconsistent with other studies. Machida et al. [33] found that older people or those with health conditions were more willing to receive vaccination. However, our findings indicate that older working-age people (Table 7 and Table 8) and those with low subjective health status (Table 5 and Table 6) were less willing and were more hesitant to receive the vaccination [33]. As negative news regarding vaccine safety in Japan and other countries is resurfacing [30,31,54], these people may have concerns about the potential side-effects and their capability of withstanding such effects. Although some recipients may experience moderate side-effects, they may take some time to recover [54,55,56,57,58,59]. As the older working-age people may have higher expected opportunity costs due to greater job-related responsibilities, they may be more hesitant to receive the vaccination. Consequently, the government should implement intervention policies that target females, the older working-age population, and those with low subjective health status.

Additionally, we found that some household, financial status, and behavioral variables influenced vaccination attitudes. First, having children had a significant positive association with people’s willingness to receive vaccination and a significantly negatively association with vaccine hesitancy. These findings may suggest that having children provides meaning in people’s lives or a sense of responsibility and accountability. Thus, these people may be more willing to receive vaccination. Because of these significant effects of having children, further studies should explore children’s characteristics in detail, such as age, number of children in the household, and so on, as they may influence attitudes toward COVID-19 vaccination.

Second, we found that financial status variables such as household income, household assets, and financial literacy significantly affect attitudes toward COVID-19 vaccination. Our findings on the income effect are consistent with other studies in Japan [33] and in other countries [12,60]. Regardless, due to the insignificant relationship between household income and vaccine hesitancy, we argue that the government should not implement policies targeting people in low-income households. The government should instead enact policies targeting people living in households with low asset value, as our findings indicate that households with more assets were more willing to receive vaccination and were less likely to be hesitant toward vaccination. The government should also introduce financial literacy improvement policies, as we found that people with better financial literacy were less likely to have vaccine hesitancy. This finding is consistent with other studies reporting that financial literacy discourages irrational behaviors [49,50,51] and is positively associated with health literacy [61].

Lastly, we found that behavioral variables such as future anxiety and a myopic view of the future affected attitudes toward vaccination. Intrinsically, people with anxiety are risk-averse; they prefer anticipated risk to unanticipated risk. Since it is easier to anticipate the risk of COVID-19 vaccination than to anticipate the risk of COVID-19 infection, we found that people with anxiety about the future were more willing to receive vaccination and were less likely to be hesitant. Meanwhile, people with a myopic view of the future may not calculate risks. We found that they were more likely to have vaccine hesitancy.

Overall, our findings that people with certain demographic, socioeconomic, and psychological backgrounds were hesitant to take the COVID-19 vaccine has important policy implications. Since the government must achieve herd immunity, it must demonstrate that the risk of COVID-19 infection is greater than the risk of COVID-19 vaccination. Chou and Budenz [62] emphasized on the evidence-based communication strategies taking peoples’ emotion into considerations to address vaccine hesitancy and foster vaccine confidence. The government may also need to establish a more effective public dialogue to ensure a general understanding of the COVID-19 situation. However, findings of this study should be generalized cautiously for other countries. Since willingness or hesitancy to take vaccine depends on several factors including behavioral and social aspects, the contributing factors could differ country-wise.

This study had some limitations. Since there is an unequal household income distribution in the internet penetration rate [63], our survey respondents may live in households with a higher income than the average Japanese household. However, the Statistics Bureau found that the average Japanese household income (before tax) is 535,392 JPY per month or 6.42 million JPY per year [64]. This is slightly higher than our sample average income (6.34 million yen per year). Additionally, the average age and gender ratios of our sample differ from the national statistics [65]. We also excluded observations because some respondents decided not to provide sensitive data such as household income and household assets. Nevertheless, our study provides robust and consistent evidence on the willingness to receive vaccination after the vaccine rollout began in Japan.

## 5. Conclusions

The COVID-19 pandemic had unprecedented effects on all aspects of life. Most countries have already started vaccination programs to contain the effects of the pandemic and to restore some normalcy to human life. However, several incidents of side-effects and the issue of vaccine effectiveness have possibly affected public acceptance of the COVID-19 vaccines. In this situation, we conducted a study to examine Japanese peoples’ willingness to take one of the vaccines using a nationwide survey in Japan in February 2021. The sample period follows the Japanese government’s initial phase of the COVID-19 vaccination program.

Our investigation showed that Japanese people were moderately willing to take a vaccine; 47% of the respondents were willing to take a vaccine once it is available, while 22% of respondents were unwilling and another 31% of respondents remained indecisive. The results further implied that 53% of respondents were hesitant to take one of the vaccines, which would be a significant enough proportion of the population to make health authorities concerned about the perceived success of the vaccination program. Comparing our results with those of previous studies seems to suggest that the willingness to receive vaccination and vaccine hesitancy vary over time. Although previous studies [23,33] suggested that some characteristics influence the willingness to receive vaccination and vaccine hesitancy, our findings suggest somewhat different implications. As negative news about the vaccines emerged in Japan, we found that people became less willing to receive a vaccination and became more hesitant. Our estimations showed that variables such as gender, age, subjective health status, children, household income, household assets, financial literacy, future anxiety, and myopic view of the future were associated with willingness to take a COVID-19 vaccine.

Because the factors associated with the willingness to take a vaccine seem to evolve over time, the Japanese government should consider the findings of our study while implementing vaccination programs, as well as for other intervention policies. Moreover, previous studies found a link between COVID-19 vaccine hesitancy and other vaccine hesitancy [23,33,39], providing grounds for a more rigorous understanding of COVID-19 vaccine hesitancy. Prior experience suggests that any negative media coverage on COVID-19 vaccines can lead to persistent vaccine hesitancy, leading to more substantial vaccine refusal in Japan. Thus, the government should target specific hesitant groups and devise appropriate motivational programs.

## Figures and Tables

**Table 1:** Variable definitions.

Variable	Definition
**Dependent Variables**	
Willingness to take vaccine	Ordinal variable: 1 = It does not hold true at all for you; 2 = It is not so true for you; 3 = It is either true or not true; 4 = It is rather true for you; 5 = It is particularly true for you for the statement “Once the Covid-19 vaccination becomes available with free of charge, I will take it soon.”
Vaccine hesitancy	Binary variable: 1 = Respondent selected 1, 2, or 3 for the statement “Once the Covid-19 vaccination becomes available with free of charge, I will take it soon,” and 0 = Otherwise.
**Explanatory Variables**	
Male *	Binary variable: 1 = Male and 0 = Female
Age *	Continuous variable: Respondent’s age
Age squared *	Continuous variable: Respondent’s age squared
University degree *	Binary variable: 1 = Obtained university degree and 0 = Otherwise
Living in central area *	Binary variable: 1 = Living in the Kanto (around Tokyo metropolis) and Kinki (around Osaka metropolis) areas and 0 = Otherwise
Marriage	Binary variable: 1 = Currently married and 0 = Otherwise
Children *	Binary variable: 1 = Respondent has child/children and 0 = Otherwise
Living alone	Binary variable: 1 = Living alone and 0 = Otherwise
Employed	Binary variable: 1 = Respondent is employed and 0 = otherwise
Household income	Continuous variable: Annual earned income before taxes and with bonuses of entire household in 2020 (unit: JPY)
Log of household income	Log of household income
Household assets	Continuous variable: Balance of financial assets (savings, stocks, bonds, insurance, etc.) of entire household (unit: JPY)
Log of household assets	Log of household assets
Financial literacy *	Continuous variable: Average score of correct answers from the three financial literacy questions
Subjective health status	Ordinal variable: 1 = It does not hold true at all for you; 2 = It is not so true for you; 3 = Neither true nor not true; 4 = It is rather true for you; 5 = It is particularly true for you for the statement “I am now healthy and was generally healthy in the last 1 year.”
Future anxiety	Ordinal variable: 1 = It does not hold true at all for you; 2 = It is not so true for you; 3 = Neither true nor not true; 4 = It is rather true for you; 5 = It is particularly true for you for the statement “I have anxieties about my life after I am 65 years old (for those who are already aged 65 or above, ‘life in the future’).”
Level of risk preference	Continuous variable: Percentage score from the question “Usually when you go out, how high does the probability of rain have to be before you take an umbrella?”
Myopic view of the future	Ordinal variable: 1 = Completely disagree; 2 = Disagree; 3 = Neither agree nor disagree; 4 = Agree; 5 = Completely agree for the statement “Since the future is uncertain, it is a waste to think about it.”

Note: * indicates data from 2020 wave.

**Table 2:** Descriptive statistics.

Variable	Mean	Standard Deviation (SD)	Min	Max
**Dependent Variables**				
Willingness to take vaccine	3.41	1.24	1	5
Vaccine hesitancy	0.53	0.50	0	1
**Explanatory Variables**				
Male	0.65	0.48	0	1
Age	50.32	13.83	21	86
Age squared	2723.05	1411.23	441	7396
University degree	0.62	0.49	0	1
Living in central area	0.61	0.49	0	1
Marriage	0.66	0.47	0	1
Children	0.57	0.49	0	1
Living alone	0.20	0.40	0	1
Employed	0.64	0.48	0	1
Household income	6,338,702	4,095,128	500,000	21,000,000
Log of household income	15.43	0.76	13.12	16.86
Household assets	19,800,000	29,100,000	1,250,000	125,000,000
Log of household assets	15.85	1.43	14.04	18.64
Financial literacy	0.65	0.36	0	1
Subjective health status	3.24	1.09	1	5
Future anxiety	3.71	1.14	1	5
Level of risk preference	0.46	0.22	0	1
Myopic view of the future	2.69	1.02	1	5
**Observations**	**4253**			

**Table 3:** Distribution of willingness to take vaccine by age group.

**Willingness to Take Vaccine**	**Age**					**Total**
	**21–30**	**31–40**	**41–50**	**51–60**	**≥61**	
1	41	66	121	79	65	372
	9.83%	9.24%	11.37%	8.14%	5.97%	8.75%
2	77	130	150	123	85	565
	18.47%	18.21%	14.10%	12.68%	7.81%	13.28%
3	136	249	386	323	239	1333
	32.61%	34.87%	36.28%	33.30%	21.97%	31.34%
4	87	141	209	189	289	915
	20.86%	19.75%	19.64%	19.48%	26.56%	21.51%
5	76	128	198	256	410	1068
	18.23%	17.93%	18.61%	26.39%	37.68%	25.11%
Total	417	714	1064	970	1088	4253
	100.00%	100.00%	100.00%	100.00%	100.00%	100.00%

**Table 4:** Distribution of vaccine hesitancy by age group.

Vaccine Hesitancy	Age					Total
	21–30	31–40	41–50	51–60	≥61	
0	163	269	407	445	699	1983
	39.09%	37.68%	38.25%	45.88%	64.25%	46.63%
1	254	445	657	525	389	2270
	60.91%	62.32%	61.75%	54.12%	35.75%	53.37%
Total	417	714	1064	970	1088	4253
	100.00%	100.00%	100.00%	100.00%	100.00%	100.00%

**Table 5:** Ordered probit model regression results (full sample analysis).

Variable	Dependent Variable: Willingness to Take Vaccine			
	Model 1.1	Model 1.2	Model 1.3	Model 1.4
Male	0.133 ***	0.141 ***	0.165 ***	0.166 ***
	(0.0402)	(0.0409)	(0.0415)	(0.0414)
Age	−0.0450 ***	−0.0486 ***	−0.0474 ***	−0.0473 ***
	(0.00839)	(0.00844)	(0.00845)	(0.00847)
Age squared	0.000566 ***	0.000589 ***	0.000579 ***	0.000577 ***
	(8.41 × 10^−5^)	(8.46 × 10^−5^)	(8.49 × 10^−5^)	(8.49 × 10^−5^)
University degree	0.0828 **	0.0311	0.0288	0.0229
	(0.0355)	(0.0372)	(0.0373)	(0.0375)
Living in central area	0.0325	0.0156	0.0214	0.0175
	(0.0339)	(0.0341)	(0.0341)	(0.0342)
Marriage	0.0680	0.0444	0.0434	0.0406
	(0.0532)	(0.0545)	(0.0544)	(0.0546)
Children	0.184 ***	0.180 ***	0.182 ***	0.182 ***
	(0.0436)	(0.0439)	(0.0438)	(0.0438)
Living alone	0.0126	0.0437	0.0418	0.0404
	(0.0568)	(0.0574)	(0.0574)	(0.0575)
Employed	0.0804 **	0.0362	0.0233	0.0231
	(0.0400)	(0.0425)	(0.0426)	(0.0426)
Log of household income		0.0677 **	0.0609 **	0.0606 **
		(0.0289)	(0.0292)	(0.0292)
Log of household assets		0.0234	0.0337 **	0.0309 **
		(0.0144)	(0.0147)	(0.0148)
Financial literacy		0.105 **	0.101 **	0.0919 *
		(0.0498)	(0.0498)	(0.0499)
Subjective health status			0.104 ***	0.105 ***
			(0.0171)	(0.0171)
Future anxiety			0.0666 ***	0.0640 ***
			(0.0170)	(0.0171)
Level of risk preference				−0.0817
				(0.0763)
Myopic view of the future				−0.0296
				(0.0183)
/cut1	−1.765 ***	−0.478	0.191	0.00789
	(0.204)	(0.429)	(0.448)	(0.459)
/cut2	−1.165 ***	0.123	0.795 *	0.611
	(0.203)	(0.429)	(0.447)	(0.458)
/cut3	−0.276	1.014 **	1.693 ***	1.510 ***
	(0.202)	(0.429)	(0.447)	(0.458)
/cut4	0.337 *	1.629 ***	2.315 ***	2.132 ***
	(0.202)	(0.429)	(0.448)	(0.458)
Observations	4253	4253	4253	4253
Log pseudolikelihood	−6332	−6321	−6291	−6289
Wald chi^2^	270.7	288.7	342	344.5
*p*-Value	0	0	0	0
Pseudo R^2^	0.0221	0.0238	0.0284	0.0287

Note: Robust standard errors are in parentheses; *** *p* < 0.01, ** *p* < 0.05, * *p* < 0.1.

**Table 6:** Probit model regression results (full sample analysis).

Variable	Dependent Variable: Vaccine Hesitancy			
	Model 2.1	Model 2.2	Model 2.3	Model 2.4
Male	−0.109 **	−0.118 **	−0.144 ***	−0.144 ***
	(0.0487)	(0.0497)	(0.0502)	(0.0503)
Age	0.0629 ***	0.0681 ***	0.0660 ***	0.0656 ***
	(0.0103)	(0.0104)	(0.0104)	(0.0104)
Age squared	−0.000775 ***	−0.000806 ***	−0.000787 ***	−0.000783 ***
	(0.000102)	(0.000103)	(0.000104)	(0.000104)
University degree	−0.108 **	−0.0386	−0.0346	−0.0284
	(0.0423)	(0.0448)	(0.0451)	(0.0453)
Living in central area	−0.0167	0.00561	−0.00194	0.00122
	(0.0409)	(0.0412)	(0.0414)	(0.0416)
Marriage	−0.0815	−0.0603	−0.0597	−0.0549
	(0.0630)	(0.0646)	(0.0648)	(0.0650)
Children	−0.166 ***	−0.166 ***	−0.165 ***	−0.165 ***
	(0.0518)	(0.0522)	(0.0523)	(0.0524)
Living alone	−0.00911	−0.0457	−0.0425	−0.0407
	(0.0667)	(0.0679)	(0.0685)	(0.0685)
Employed	−0.103 **	−0.0567	−0.0433	−0.0427
	(0.0483)	(0.0517)	(0.0520)	(0.0521)
Log of household income		−0.0647 *	−0.0554	−0.0548
		(0.0345)	(0.0349)	(0.0349)
Log of household assets		−0.0409 **	−0.0510 ***	−0.0478 ***
		(0.0168)	(0.0174)	(0.0175)
Financial literacy		−0.147 **	−0.145 **	−0.132 **
		(0.0610)	(0.0613)	(0.0615)
Subjective health status			−0.127 ***	−0.129 ***
			(0.0187)	(0.0187)
Future anxiety			−0.0661 ***	−0.0626 ***
			(0.0188)	(0.0189)
Level of risk preference				0.0635
				(0.0900)
Myopic view of the future				0.0385 *
				(0.0200)
Constant	−0.604 **	0.875 *	1.611 ***	1.404 **
	(0.247)	(0.517)	(0.544)	(0.554)
Observations	4253	4253	4253	4253
Log pseudolikelihood	−2803	−2789	−2761	−2759
Wald chi^2^	255.1	278.7	323.9	326.6
*p*-Value	0	0	0	0
Pseudo R^2^	0.0462	0.0506	0.0602	0.0609

Note: Robust standard errors are in parentheses; *** *p* < 0.01, ** *p* < 0.05, * *p* < 0.1.

**Table 7:** Ordered probit model regression results (subsample analysis: age).

Variable	Dependent Variable: Willingness to Take Vaccine							
	Sub-Sample: Age <65				Sub-Sample: Age ≥65			
	Model 3.1	Model 3.2	Model 3.3	Model 3.4	Model 4.1	Model 4.2	Model 4.3	Model 4.4
Male	0.130 ***	0.137 ***	0.164 ***	0.164 ***	0.156	0.202 *	0.227 *	0.231 *
	(0.0435)	(0.0442)	(0.0449)	(0.0449)	(0.120)	(0.123)	(0.123)	(0.123)
Age	−0.0535 ***	−0.0575 ***	−0.0581 ***	−0.0571 ***	0.0292	−0.0346	−0.0386	−0.0280
	(0.0135)	(0.0136)	(0.0136)	(0.0136)	(0.267)	(0.264)	(0.268)	(0.269)
Age squared	0.000666 ***	0.000700 ***	0.000711 ***	0.000699 ***	−7.14 × 10^−5^	0.000348	0.000369	0.000296
	(0.000150)	(0.000152)	(0.000152)	(0.000152)	(0.00185)	(0.00183)	(0.00186)	(0.00187)
University degree	0.0737 *	0.0242	0.0213	0.0144	0.0985	0.0389	0.0317	0.0337
	(0.0390)	(0.0410)	(0.0412)	(0.0414)	(0.0915)	(0.0944)	(0.0942)	(0.0942)
Living in central area	0.0152	−0.000528	0.00702	0.00335	0.130	0.0990	0.0979	0.0935
	(0.0372)	(0.0374)	(0.0374)	(0.0375)	(0.0832)	(0.0831)	(0.0834)	(0.0841)
Marriage	0.0802	0.0478	0.0432	0.0394	0.104	0.0735	0.0865	0.0885
	(0.0562)	(0.0579)	(0.0578)	(0.0581)	(0.180)	(0.180)	(0.180)	(0.179)
Children	0.145 ***	0.138 ***	0.144 ***	0.144 ***	0.456 ***	0.452 ***	0.450 ***	0.449 ***
	(0.0464)	(0.0467)	(0.0466)	(0.0466)	(0.132)	(0.132)	(0.133)	(0.133)
Living alone	0.00653	0.0374	0.0356	0.0347	0.117	0.148	0.143	0.145
	(0.0601)	(0.0608)	(0.0609)	(0.0610)	(0.198)	(0.200)	(0.199)	(0.199)
Employed	0.0869 *	0.0350	0.0174	0.0173	0.0645	0.0407	0.0411	0.0419
	(0.0453)	(0.0481)	(0.0482)	(0.0482)	(0.0919)	(0.0990)	(0.0994)	(0.0993)
Log of household income		0.0853 ***	0.0785 **	0.0781 **		0.0353	0.0225	0.0251
		(0.0314)	(0.0317)	(0.0318)		(0.0783)	(0.0783)	(0.0789)
Log of household assets		0.00953	0.0194	0.0166		0.0660 *	0.0638 *	0.0644 *
		(0.0159)	(0.0163)	(0.0164)		(0.0337)	(0.0357)	(0.0362)
Financial literacy		0.1000 *	0.101 *	0.0908 *		0.177	0.169	0.171
		(0.0538)	(0.0539)	(0.0539)		(0.136)	(0.136)	(0.136)
Subjective health status			0.113 ***	0.115 ***			0.0621	0.0623
			(0.0191)	(0.0191)			(0.0395)	(0.0395)
Future anxiety			0.0819 ***	0.0782 ***			−0.0161	−0.0156
			(0.0185)	(0.0187)			(0.0435)	(0.0434)
Level of risk preference				−0.0794				−0.0703
				(0.0826)				(0.200)
Myopic view of the future				−0.0356 *				0.0163
				(0.0203)				(0.0425)
/cut1	−1.989 ***	−0.661	0.0589	−0.129	0.865	0.157	−0.0795	0.363
	(0.295)	(0.503)	(0.519)	(0.529)	(9.588)	(9.559)	(9.741)	(9.794)
/cut2	−1.370 ***	−0.0407	0.682	0.494	1.336	0.630	0.393	0.835
	(0.294)	(0.503)	(0.519)	(0.529)	(9.583)	(9.554)	(9.735)	(9.789)
/cut3	−0.442	0.890 *	1.622 ***	1.435 ***	2.000	1.297	1.061	1.504
	(0.293)	(0.503)	(0.519)	(0.529)	(9.583)	(9.554)	(9.735)	(9.789)
/cut4	0.148	1.482 ***	2.222 ***	2.035 ***	2.721	2.025	1.792	2.235
	(0.293)	(0.503)	(0.520)	(0.529)	(9.583)	(9.554)	(9.736)	(9.789)
Observations	3485	3485	3485	3485	768	768	768	768
Log pseudolikelihood	−5258	−5249	−5217	−5215	−1052	−1048	−1046	−1046
Wald chi^2^	113.8	129.3	182.2	185.1	28.11	38.54	41.27	42.26
*p*-Value	0	0	0	0	0.000914	0.000125	0.000161	0.000360
Pseudo R^2^	0.0104	0.0120	0.0180	0.0184	0.0141	0.0187	0.0203	0.0204

Note: Robust standard errors are in parentheses; *** *p* < 0.01, ** *p* < 0.05, * *p* < 0.1.

**Table 8:** Probit model regression results (sub-sample analysis: age).

Variable	Dependent Variable: Vaccine Hesitancy							
	Sub-Sample: Age <65				Sub-Sample: Age ≥65			
	Model 5.1	Model 5.2	Model 5.3	Model 5.4	Model 6.1	Model 6.2	Model 6.3	Model 6.4
Male	−0.0973 *	−0.104 *	−0.133 **	−0.132 **	−0.198	−0.266 *	−0.289 **	−0.298 **
	(0.0530)	(0.0540)	(0.0548)	(0.0549)	(0.138)	(0.142)	(0.142)	(0.143)
Age	0.0655 ***	0.0710 ***	0.0710 ***	0.0693 ***	0.0862	0.185	0.180	0.157
	(0.0163)	(0.0164)	(0.0166)	(0.0166)	(0.301)	(0.298)	(0.304)	(0.305)
Age squared	−0.000803 ***	−0.000843 ***	−0.000851 ***	−0.000832 ***	−0.000738	−0.00139	−0.00135	−0.00119
	(0.000181)	(0.000182)	(0.000184)	(0.000184)	(0.00208)	(0.00206)	(0.00210)	(0.00211)
University degree	−0.0996 **	−0.0303	−0.0251	−0.0182	−0.107	−0.0365	−0.0247	−0.0277
	(0.0464)	(0.0493)	(0.0498)	(0.0500)	(0.107)	(0.111)	(0.111)	(0.112)
Living in central area	−0.000356	0.0209	0.0109	0.0131	−0.125	−0.0833	−0.0812	−0.0711
	(0.0450)	(0.0453)	(0.0455)	(0.0458)	(0.0999)	(0.101)	(0.101)	(0.102)
Marriage	−0.108	−0.0765	−0.0713	−0.0642	0.0137	0.0525	0.0528	0.0512
	(0.0668)	(0.0688)	(0.0691)	(0.0693)	(0.211)	(0.212)	(0.213)	(0.213)
Children	−0.109 **	−0.104 *	−0.109 *	−0.108 *	−0.549 ***	−0.556 ***	−0.558 ***	−0.554 ***
	(0.0554)	(0.0559)	(0.0561)	(0.0562)	(0.148)	(0.149)	(0.149)	(0.150)
Living alone	−0.00270	−0.0399	−0.0380	−0.0369	−0.0518	−0.0695	−0.0426	−0.0421
	(0.0703)	(0.0716)	(0.0725)	(0.0725)	(0.230)	(0.233)	(0.235)	(0.235)
Employed	−0.122 **	−0.0647	−0.0451	−0.0444	−0.0625	−0.0555	−0.0596	−0.0611
	(0.0548)	(0.0587)	(0.0591)	(0.0592)	(0.112)	(0.119)	(0.120)	(0.120)
Log of household income		−0.0883 **	−0.0793 **	−0.0787 **		0.0135	0.0343	0.0295
		(0.0374)	(0.0381)	(0.0381)		(0.0955)	(0.0959)	(0.0961)
Log of household assets		−0.0242	−0.0347 *	−0.0314		−0.105 ***	−0.0921 **	−0.0934 **
		(0.0187)	(0.0193)	(0.0194)		(0.0388)	(0.0419)	(0.0423)
Financial literacy		−0.147 **	−0.151 **	−0.137 **		−0.210	−0.213	−0.219
		(0.0662)	(0.0667)	(0.0670)		(0.162)	(0.163)	(0.164)
Subjective health status			−0.143 ***	−0.146 ***			−0.0498	−0.0501
			(0.0208)	(0.0208)			(0.0452)	(0.0452)
Future anxiety			−0.0882 ***	−0.0827 ***			0.0567	0.0561
			(0.0207)	(0.0208)			(0.0494)	(0.0494)
Level of risk preference				0.0443				0.157
				(0.0983)				(0.234)
Myopic view of the future				0.0490 **				−0.0310
				(0.0222)				(0.0478)
Constant	−0.678 *	0.894	1.741 ***	1.532 **	−2.084	−4.144	−4.593	−3.647
	(0.354)	(0.602)	(0.628)	(0.637)	(10.85)	(10.84)	(11.07)	(11.15)
Observations	3485	3485	3485	3485	768	768	768	768
Log pseudolikelihood	−2328	−2319	−2287	−2284	−466.8	−460.6	−459	−458.6
Wald chi^2^	81.68	100.8	159.3	163.6	26.73	38.50	41.43	42.17
*p*-Value	0	0	0	0	0.00155	0.000127	0.000152	0.000372
Pseudo R^2^	0.0172	0.0214	0.0347	0.0358	0.0278	0.0405	0.0439	0.0448

Note: Robust standard errors are in parentheses; *** *p* < 0.01, ** *p* < 0.05, * *p* < 0.1.

**Table 9:** Ordered probit model regression results (subsample analysis: gender).

Variable	Dependent Variable: Willingness to Take Vaccine							
	Sub-Sample: Female				Sub-Sample: Male			
	Model 7.1	Model 7.2	Model 7.3	Model 7.4	Model 8.1	Model 8.2	Model 8.3	Model 8.4
Age	−0.0453 ***	−0.0485 ***	−0.0444 ***	−0.0442 ***	−0.0431 ***	−0.0438 ***	−0.0435 ***	−0.0438 ***
	(0.0137)	(0.0136)	(0.0137)	(0.0137)	(0.0120)	(0.0120)	(0.0121)	(0.0121)
Age squared	0.000574 ***	0.000593 ***	0.000548 ***	0.000543 ***	0.000545 ***	0.000540 ***	0.000538 ***	0.000540 ***
	(0.000147)	(0.000147)	(0.000148)	(0.000148)	(0.000117)	(0.000117)	(0.000118)	(0.000118)
University degree	0.121 **	0.0759	0.0618	0.0581	0.0572	−0.00437	−0.00126	−0.00760
	(0.0580)	(0.0611)	(0.0613)	(0.0614)	(0.0456)	(0.0478)	(0.0481)	(0.0484)
Living in central area	−0.0248	−0.0423	−0.0372	−0.0467	0.0649	0.0476	0.0547	0.0524
	(0.0563)	(0.0568)	(0.0567)	(0.0570)	(0.0425)	(0.0427)	(0.0427)	(0.0428)
Marriage	0.0923	0.0404	0.0175	0.0176	0.0543	0.0473	0.0602	0.0557
	(0.0845)	(0.0869)	(0.0872)	(0.0874)	(0.0718)	(0.0727)	(0.0724)	(0.0728)
Children	0.176 ***	0.172 ***	0.165 **	0.168 **	0.193 ***	0.185 ***	0.189 ***	0.189 ***
	(0.0654)	(0.0661)	(0.0662)	(0.0662)	(0.0599)	(0.0603)	(0.0600)	(0.0601)
Living alone	0.0561	0.113	0.0981	0.0950	−0.0138	0.00803	0.0145	0.0135
	(0.0957)	(0.0974)	(0.0975)	(0.0976)	(0.0707)	(0.0713)	(0.0712)	(0.0713)
Employed	0.0844	0.0291	0.0102	0.0138	0.0661	0.0248	0.0112	0.00857
	(0.0622)	(0.0647)	(0.0648)	(0.0647)	(0.0579)	(0.0634)	(0.0638)	(0.0637)
Log of household income		0.114 **	0.108 **	0.108 **		0.0462	0.0395	0.0391
		(0.0460)	(0.0459)	(0.0459)		(0.0379)	(0.0385)	(0.0385)
Log of household assets		0.0134	0.0143	0.0112		0.0292 *	0.0452 **	0.0419 **
		(0.0255)	(0.0261)	(0.0260)		(0.0174)	(0.0179)	(0.0180)
Financial literacy		0.0339	0.0516	0.0424		0.153 **	0.134 **	0.122 *
		(0.0788)	(0.0790)	(0.0792)		(0.0648)	(0.0649)	(0.0649)
Subjective health status			0.104 ***	0.106 ***			0.104 ***	0.105 ***
			(0.0285)	(0.0285)			(0.0215)	(0.0215)
Future anxiety			0.0386	0.0369			0.0812 ***	0.0780 ***
			(0.0276)	(0.0279)			(0.0215)	(0.0216)
Level of risk preference				−0.209				−0.0378
				(0.140)				(0.0909)
Myopic view of the future				−0.0132				−0.0380 *
				(0.0309)				(0.0228)
/cut1	−1.844 ***	−0.0421	0.422	0.216	−1.819 ***	−0.672	0.104	−0.111
	(0.316)	(0.681)	(0.707)	(0.722)	(0.302)	(0.592)	(0.612)	(0.627)
/cut2	−1.139 ***	0.665	1.131	0.926	−1.289 ***	−0.142	0.638	0.422
	(0.314)	(0.679)	(0.705)	(0.720)	(0.301)	(0.592)	(0.611)	(0.626)
/cut3	−0.263	1.544 **	2.017 ***	1.813 **	−0.393	0.757	1.544 **	1.329 **
	(0.312)	(0.679)	(0.706)	(0.721)	(0.300)	(0.592)	(0.612)	(0.626)
/cut4	0.385	2.194 ***	2.673 ***	2.470 ***	0.206	1.359 **	2.152 ***	1.938 ***
	(0.312)	(0.679)	(0.707)	(0.722)	(0.300)	(0.592)	(0.612)	(0.627)
Observations	1470	1470	1470	1470	2783	2783	2783	2783
Log pseudolikelihood	−2241	−2236	−2227	−2226	−4083	−4074	−4053	−4051
Wald chi^2^	48.44	59.82	73.18	74.88	156.5	170.7	211.2	213.5
*p*-Value	8.12 × 10^−8^	1.00 × 10^−8^	2.07 × 10^−10^	5.95 × 10^−10^	0	0	0	0
Pseudo R^2^	0.0114	0.0135	0.0174	0.0180	0.0199	0.0219	0.0271	0.0275

Note: Robust standard errors are in parentheses; *** *p* < 0.01, ** *p* < 0.05, * *p* < 0.1.

**Table 10:** Probit model regression results (subsample analysis: gender).

Variable	Dependent Variable: Vaccine Hesitancy							
	Sub-Sample: Female				Sub-Sample: Male			
	Model 9.1	Model 9.2	Model 9.3	Model 9.4	Model 10.1	Model 10.2	Model 10.3	Model 10.4
Age	0.0641 ***	0.0683 ***	0.0615 ***	0.0615 ***	0.0590 ***	0.0603 ***	0.0597 ***	0.0597 ***
	(0.0166)	(0.0166)	(0.0168)	(0.0169)	(0.0146)	(0.0147)	(0.0148)	(0.0148)
Age squared	−0.000794 ***	−0.000823 ***	−0.000748 ***	−0.000744 ***	−0.000732 ***	−0.000725 ***	−0.000720 ***	−0.000720 ***
	(0.000175)	(0.000175)	(0.000178)	(0.000178)	(0.000142)	(0.000143)	(0.000144)	(0.000144)
University degree	−0.159 **	−0.111	−0.0895	−0.0865	−0.0752	0.0145	0.0122	0.0198
	(0.0694)	(0.0739)	(0.0746)	(0.0746)	(0.0537)	(0.0570)	(0.0574)	(0.0578)
Living in central area	−0.00856	0.0105	0.00114	0.00877	−0.0220	0.00350	−0.00451	−0.00168
	(0.0691)	(0.0695)	(0.0698)	(0.0703)	(0.0509)	(0.0513)	(0.0516)	(0.0519)
Marriage	−0.166	−0.110	−0.0809	−0.0805	−0.0512	−0.0488	−0.0633	−0.0561
	(0.101)	(0.105)	(0.105)	(0.105)	(0.0841)	(0.0853)	(0.0856)	(0.0858)
Children	−0.196 **	−0.192 **	−0.179 **	−0.181 **	−0.166 **	−0.161 **	−0.162 **	−0.163 **
	(0.0817)	(0.0828)	(0.0833)	(0.0834)	(0.0682)	(0.0687)	(0.0687)	(0.0688)
Living alone	−0.0858	−0.146	−0.121	−0.118	0.0376	0.00812	0.000328	0.00170
	(0.111)	(0.115)	(0.117)	(0.117)	(0.0838)	(0.0849)	(0.0854)	(0.0855)
Employed	−0.183 **	−0.124	−0.102	−0.105	−0.0476	−0.00290	0.0107	0.0153
	(0.0774)	(0.0810)	(0.0814)	(0.0814)	(0.0680)	(0.0748)	(0.0755)	(0.0755)
Log of household income		−0.128 **	−0.118 **	−0.117 **		−0.0415	−0.0340	−0.0335
		(0.0576)	(0.0582)	(0.0582)		(0.0443)	(0.0449)	(0.0449)
Log of household assets		−0.0102	−0.00910	−0.00682		−0.0541 ***	−0.0703 ***	−0.0657 ***
		(0.0308)	(0.0314)	(0.0315)		(0.0201)	(0.0210)	(0.0212)
Financial literacy		−0.0498	−0.0764	−0.0697		−0.214 ***	−0.196 **	−0.178 **
		(0.0989)	(0.0993)	(0.0997)		(0.0781)	(0.0785)	(0.0789)
Subjective health status			−0.154 ***	−0.155 ***			−0.113 ***	−0.115 ***
			(0.0323)	(0.0324)			(0.0231)	(0.0232)
Future anxiety			−0.0392	−0.0379			−0.0797 ***	−0.0748 ***
			(0.0322)	(0.0324)			(0.0232)	(0.0234)
Level of risk preference				0.170				0.0363
				(0.168)				(0.107)
Myopic view of the future				0.00831				0.0544 **
				(0.0344)				(0.0247)
Constant	−0.477	1.476 *	2.058 **	1.891 **	−0.728 **	0.731	1.543 **	1.254 *
	(0.378)	(0.855)	(0.903)	(0.915)	(0.365)	(0.700)	(0.729)	(0.744)
Observations	1470	1470	1470	1470	2783	2783	2783	2783
Log pseudolikelihood	−955.1	−951.3	−939.3	−938.8	−1845	−1833	−1816	−1814
Wald chi^2^	57.71	65.19	85.72	85.78	154.8	177.9	202.7	206.6
*p*-Value	1.31 × 10^−9^	9.93 × 10^−10^	0	0	0	0	0	0
Pseudo R^2^	0.0292	0.0330	0.0452	0.0458	0.0433	0.0496	0.0583	0.0596

Note: Robust standard errors are in parentheses; *** *p* < 0.01, ** *p* < 0.05, * *p* < 0.1.

## Data Availability

Data are available on request.

## Supplementary Materials

The following are available online at https://www.mdpi.com/article/10.3390/ijerph18094864/s1. Table S1: Correlation matrix.
