# Supplementary material for: Willing or Hesitant? A Socioeconomic Study on the Potential Acceptance of COVID-19 Vaccine in Japan
Source: Int J Environ Res Public Health. 2021 May 2;18(9):4864. doi: 10.3390/ijerph18094864 (PMC8125588; doi:10.3390/ijerph18094864)
Supplement: Supplementary file 1 [file ijerph-18-04864-s001.zip › ijerph-1207333-supplementary.pdf]

Table: Correlation matrix

Variables	Willingness to take vaccine	Vaccine hesitancy	Male	Age	Age squared	University degree	Live in central area	Marriage	Children
Willingness to take vaccine	1								
Vaccine hesitancy	-0.8518	1							
Male	0.1266	-0.1094	1						
Age	0.1967	-0.1958	0.3299	1					
Age squared	0.2074	-0.2094	0.3127	0.9891	1				
University degree	0.0631	-0.0618	0.2159	0.0107	0.0128	1			
Live in central area	0.0228	-0.016	0.0505	0.0573	0.0511	0.1323	1		
Marriage	0.1112	-0.1008	0.05	0.2167	0.2139	0.0642	0.0121	1	
Children	0.1557	-0.1385	0.0955	0.367	0.362	0.0144	-0.0391	0.5886	1
Live alone	-0.0658	0.0583	-0.0256	-0.1304	-0.124	-0.0111	0.0088	-0.6709	-0.4305
Employed	-0.0137	0.024	0.2974	-0.2518	-0.2858	0.1711	0.0122	-0.1008	-0.0989
Log of household income	0.082	-0.0711	0.0663	-0.0292	-0.0478	0.233	0.104	0.3581	0.2408
Log of household assets	0.1157	-0.1247	0.0909	0.2975	0.2911	0.2268	0.1244	0.1186	0.1157
Financial literacy	0.0982	-0.1021	0.2444	0.2263	0.2146	0.278	0.0851	0.0446	0.0537
Subjective health	0.1166	-0.1222	-0.0098	0.0169	0.0271	0.0571	-0.0044	0.0576	0.0564
Future anxiety	-0.0168	0.0305	-0.1169	-0.166	-0.1758	-0.1051	-0.0552	-0.1028	-0.1189
Level of risk preference	-0.0388	0.0332	-0.0194	-0.1174	-0.1121	-0.0891	-0.1302	0.0131	-0.0095
Myopic view of the future	-0.049	0.0527	-0.048	-0.0175	-0.0183	-0.107	-0.024	-0.0716	-0.0403

Table: Correlation matrix (continue)

Variables	Live alone	Employed	Log of household income	Log of household assets	Financial literacy	Subjective health	Future anxiety	Level of risk preference	Myopic view of the future
Live alone	1								
Employed	0.116	1							
Log of household income	-0.3173	0.3168	1						
Log of household assets	-0.1234	-0.002	0.4008	1					
Financial literacy	-0.0161	0.054	0.1512	0.2991	1				
Subjective health	-0.0122	0.0267	0.1118	0.0855	0.0255	1			
Future anxiety	0.0428	0.0246	-0.1578	-0.3094	-0.0966	-0.1173	1		
Level of risk preference	-0.012	0.0226	-0.0336	-0.1116	-0.0899	0.0124	0.0462	1	
Myopic view of the future	0.0454	-0.032	-0.0803	-0.1112	-0.1327	0.0377	-0.0555	0.0367	1

Table: Variance Inflation Factor (VIF) test

Variables	VIF	1/VIF
Marriage	2.4	0.416724
Live alone	1.9	0.525766
Log of household income	1.76	0.569352
Children	1.73	0.57788
Age	1.72	0.582403
Log of household assets	1.57	0.636696
Employed	1.53	0.654954
Male	1.46	0.68496
Financial literacy	1.23	0.810928
University degree	1.21	0.82433
Future anxiety	1.15	0.866086
Live in central area	1.05	0.949535
Myopic view of the future	1.05	0.955529
Level of risk preference	1.04	0.95712
**Mean VIF**	**1.46**	
